# The Role of Membrane Capacitance in Cardiac Impulse Conduction: An Optogenetic Study With Non-excitable Cells Coupled to Cardiomyocytes

**DOI:** 10.3389/fphys.2020.00194

**Published:** 2020-03-26

**Authors:** Stefano Andrea De Simone, Sarah Moyle, Andrea Buccarello, Christian Dellenbach, Jan Pavel Kucera, Stephan Rohr

**Affiliations:** ^1^Laboratory of Cellular Optics II, Department of Physiology, University of Bern, Bern, Switzerland; ^2^Integrative Cardiac Bioelectricity Group, Department of Physiology, University of Bern, Bern, Switzerland

**Keywords:** heart, fibroblasts, myofibroblasts, conduction velocity, membrane capacitance, optogenetics, computer modeling, arrhythmia

## Abstract

Non-excitable cells (NECs) such as cardiac myofibroblasts that are electrotonically coupled to cardiomyocytes affect conduction velocity (θ) by representing a capacitive load (CL: increased membrane to be charged) and a resistive load (RL: partial depolarization of coupled cardiomyocytes). In this study, we untangled the relative contributions of both loading modalities to NEC-dependent arrhythmogenic conduction slowing. Discrimination between CL and RL was achieved by reversibly removing the RL component by light activation of the halorhodopsin-based hyperpolarizing membrane voltage actuator eNpHR3.0-eYFP (enhanced yellow fluorescent protein) expressed in communication-competent fibroblast-like NIH3T3 cells (3T3_*HR*_ cells) that served as a model of coupled NECs. Experiments were conducted with strands of neonatal rat ventricular cardiomyocytes coated at increasing densities with 3T3_*HR*_ cells. Impulse conduction along preparations stimulated at 2.5 Hz was assessed with multielectrode arrays. The relative density of 3T3_*HR*_ cells was determined by dividing the area showing eYFP fluorescence by the area covered with cardiomyocytes [coverage factor (CF)]. Compared to cardiomyocytes, 3T3_*HR*_ cells exhibited a depolarized membrane potential (−34 mV) that was shifted to −104 mV during activation of halorhodopsin. Without illumination, 3T3_*HR*_ cells slowed θ along the preparations from ∼330 mm/s (control cardiomyocyte strands) to ∼100 mm/s (CF = ∼0.6). Illumination of the preparation increased the electrogram amplitudes and induced partial recovery of θ at CF > 0.3. Computer simulations demonstrated that the θ deficit observed during illumination was attributable in full to the CL represented by coupled 3T3_*HR*_ cells with θ showing a power-law relationship to capacitance with an exponent of −0.78 (simulations) and −0.99 (experiments). The relative contribution of CL and RL to conduction slowing changed as a function of CF with CL dominating at CF ≤ ∼0.3, both mechanisms being equally important at CF = ∼0.5, and RL dominating over CL at CF > 0.5. The finding that RL did not affect θ at CFs ≤ 0.3 is explained by the circumstance that, at the respective moderate levels of cardiomyocyte depolarization, supernormal conduction stabilized propagation. The findings provide experimental estimates for the dependence of θ on membrane capacitance in general and suggest that the myocardium can absorb moderate numbers of electrotonically coupled NECs without showing substantial alterations of θ.

## Introduction

Heart rhythm disorders are frequent complications of cardiac disease. The initiation of reentrant arrhythmias such as flutter and fibrillation results from slow conduction of the cardiac action potential and conduction block (Kleber and Rudy, 2004). Classically, the velocity of conduction is determined primarily by the density and kinetics of voltage-gated channels carrying inward currents, as well as by the level of gap junctional coupling between cardiomyocytes (CMCs) (Shaw and Rudy, 1997; Rohr et al., 1998). More recently, it was shown both *in vitro* and *in vivo* that conduction velocity (θ) can be modulated by non-excitable cells (NECs) such as myofibroblasts and macrophages that are coupled to CMCs by gap junctions (Rohr, 2009; Hulsmans et al., 2017).

Electrotonic coupling of NECs to CMCs slows impulse conduction based on two main mechanisms: (1) NECs like myofibroblasts exhibit a reduced (less negative) membrane potential (*V*_*m*_) compared to CMCs (Salvarani et al., 2017). Accordingly, they induce partial CMC depolarization upon establishment of heterocellular electrotonic coupling. The extent of CMC depolarization depends on the difference between the membrane potentials of the two cell types (Δ*V*_*m*_) and the relative magnitudes of the CMC membrane resistance, the NEC membrane resistance, and the gap junctional conductance (Jousset et al., 2016; Kucera et al., 2017). The NEC-dependent reduction of the resting membrane potential (RMP) of coupled CMCs induces sodium channel inactivation and hence reduces θ (Miragoli et al., 2006). The effect of such “resistive loading,” that is, the reduction of the RMP of CMCs by coupled, less polarized cells, has been investigated before using bioengineered strands of CMCs that were coated with cardiac myofibroblasts known to establish heterocellular electrotonic coupling. With increasing myofibroblast density, θ was reported to first slightly increase and then monotonically decrease, thereby reproducing the phenomenon of supernormal conduction that characterizes the response of θ to increasing CMC depolarization as induced, for example, by a gradual increasing extracellular potassium concentration (Kagiyama et al., 1982; Shaw and Rudy, 1997; Rohr et al., 1998; Miragoli et al., 2006; Jacquemet and Henriquez, 2008). (2) Even if NECs were to display a *V*_*m*_ similar to the RMP of CMCs, and hence, sodium-channel availability would not be compromised, electrotonic coupling between the two cell types would still be expected to slow conduction because the membrane capacitance of NECs will be charged during activation of coupled CMCs, which results in a reduction of the amount of depolarizing current available for an efficient downstream depolarization of CMCs as shown before in computer simulations (Jacquemet and Henriquez, 2008).

By contrast to the established role of resistive loading of CMCs by coupled NECs in conduction slowing, experimental data that characterize the contribution of capacitive loading to conduction slowing are, to our knowledge, still lacking. In excitable cells, the membrane capacitance (*C*_*m*_) delays the onset of the action potential upstroke due to a prolongation of the foot potential. This results in slowing of conduction, with θ generally being assumed to show an inverse proportionality to *C*_*m*_ in cardiac tissue (Matsumoto and Tasaki, 1977). The same proportionality is expected to govern conduction in nerve fibers (Hartline and Colman, 2007). For the case of NECs being electrotonically coupled to CMCs, previous *in silico* studies predicted θ to be inversely proportional to the square root of *C*_*m*_ of coupled NECs with the magnitude of the effect on conduction being dependent on the coupling conductance between the two cell types (Plonsey and Barr, 2000; Jacquemet and Henriquez, 2008). However, earlier theoretical work suggests that the relationship between θ and tissue capacitance does not necessarily follow an inverse law or an inverse square root law but more generally a power law with an exponent between −1/2 and −1 and that this power-law relationship depends on the density and kinetic properties of the voltage-gated channels in addition to purely passive electrical properties (Huxley, 1959; Jack et al., 1983).

Whereas the results of previous computer simulations underline the importance of capacitive loading of CMCs by coupled NECs in proarrhythmic slowing of conduction, a lack of appropriate methodologies has precluded a direct experimental assessment of theoretical predictions in the past. This situation has markedly changed with the advent of optogenetics that we use in this study to experimentally untangle the differential contributions of capacitive versus resistive loading to conduction slowing induced by NECs coupled to CMCs. The results show that NEC-dependent capacitive loading slows θ in a ∼1/*C*_*m*_ fashion. At low NEC densities, this effect is offset by resistive loading that stabilizes θ due to supernormal conduction, whereas at higher NEC densities, both capacitive and resistive loading synergistically reduce θ. Being aware of the complex interactions between the two loading conditions is expected to contribute to the general understanding of the effects that NECs coupled to CMCs exert on cardiac impulse propagation in health and disease.

## Materials and Methods

### Production of Lentiviral Expression Vectors for the Halorhodopsin Gene eNpHR3.0

The plasmids pLenti-CMV-GFP-Puro-(658-5) (Addgene, ref. #17448) and pLenti-CaMKIIa-eNpHR3.0-EYFP-WPRE (Addgene, ref. #20946) were combined into the customized lentiviral expression vector pLenti-CMV-eNpHR3.0-Puro. This vector induced the expression of the enhanced version of halorhodopsin (HR) (eNpHR3.0) under the CMV (human cytomegalovirus) promoter with the puromycin resistance gene serving to create a 3T3 cell line stably expressing HR. Molecular cloning was performed using classical techniques and by employing the In-Fusion strategy (Takara Bio Europe, Saint-Germain-en-Laye, France).

### Lentivirus Production

The lentiviral expression vector was cotransfected in HEK293 cells with the plasmids encoding for the vesicular stomatitis virus G protein (pMD2.G, Addgene ref. #12259) and the Gag and Pol polyproteins and the Tat and Rev proteins of human immunodeficiency virus type 1 (pCMVR8.74, Addgene ref. #22036). HEK293 cells (3–6 × 10^7^ cells) were grown and transfected in Dulbecco modified Eagle medium (DMEM) (ThermoFisher, Reinach, Switzerland) supplemented with 10% fetal bovine serum (Bioswisstech Ltd., Schaffhausen, Switzerland) and 0.5 mmol/L sodium pyruvate (Merck, Buchs, Switzerland). Transfection was performed with Transit-LT1 reagent (Mirus Bio LLC, Madison, MI, United States) composed of a mixture of lipids and proteins/polyamines. After transfection, the medium was collected for 2 days and stored at 4°C until the day of ultracentrifugation (74,000 *g* for 2 h at 4°C) to isolate the viral particles. The lentiviral particles were resuspended in an appropriate volume of sterile phosphate-buffered saline and stored at −80°C. Titration was performed by transducing HEK293 cells followed by assessing the transducing units (T.U.), which ranged from 10^8^ to 10^10^ T.U./mL. Production, purification, and titration were adapted from Hewinson et al. (2013).

### Generation of 3T3_*HR*_ Cells

NIH3T3 cells (mouse embryonic fibroblast cell line) were stably transduced (puromycin selection) with HR-expressing lentiviral particles to generate a cell line that we named 3T3_*HR*_. For this purpose, NIH3T3 cells (Y. Zimmer lab, DBMR, University of Bern, Switzerland) were kept in culture medium consisting of DMEM (ThermoFisher, Reinach, Switzerland) supplemented with 10% fetal bovine serum (Bioswisstech Ltd., Schaffhausen, Switzerland), 10 U/mL penicillin, 10 μg/mL streptomycin (Bioswisstech Ltd., Schaffhausen, Switzerland), and 0.5 mmol/L sodium pyruvate (Merck, Buchs, Switzerland). During transduction, the cell culture medium was replaced by supplemented medium containing polybrene (8 μg/mL; Santa Cruz, Heidelberg, Germany) and lentiviral particles (multiplicity of infection, MOI, ranging between 3 and 8). This medium was replaced after 18 h with normal supplemented DMEM. Cells were cultured at 37°C, 5% CO_2_ in culture medium supplemented with puromycin (2 μg/mL; Santa Cruz, Heidelberg, Germany) until reaching confluency after 4 to 7 days. Thereafter, the transduced cells (3T3_*HR*_ cells) were dissociated, centrifuged (1,000 revolutions per min for 10 min), resuspended in cell culture medium containing 20% fetal bovine serum and 10% dimethyl sulfoxide at 10^6^ cells/mL, and finally stored in liquid nitrogen. For use in experiments, 3T3_*HR*_ cells were defrosted and kept in supplemented DMEM medium containing puromycin (2 μg/mL) for 1 week. The cell monolayers were dissociated using trypsin-EDTA solution (Merck, Buchs, Switzerland). The resulting cell suspension was centrifuged, and the cell pellet resuspended in supplemented cell culture medium M199 (for composition cf. below). After determination of the cell count with a hemocytometer, 3T3_*HR*_ cells were seeded at defined densities onto CMC strand preparations (see below) or on glass coverslips (25 cells/mm^2^) for patch clamp experiments.

### CMC Cell Strands

Neonatal rat ventricular CMC cultures were prepared using established protocols and following Swiss guidelines for animal experimentation under the license BE27/17 of the State Veterinary Department of the Canton of Bern (Rohr et al., 2003). In short, the ventricles of the hearts of 8 to 10 neonatal rats (Wistar, 1 day old) were minced with scissors, and the resulting tissue pieces dissociated in Hanks balanced salt solution (HBSS) (without Ca^2+^ and Mg^2+^; Bioconcept AG, Allschwil, Switzerland) that contained trypsin (0.1%; Merck, Buchs, Switzerland) and pancreatin (120 μg/mL; Merck, Buchs, Switzerland). After centrifugation, the dissociated cells were resuspended in medium M199 with Hanks salts (Merck, Buchs, Switzerland) that was supplemented with penicillin (20 U/mL; Merck, Buchs, Switzerland), vitamin B_12_ (2 μg/mL; Merck, Buchs, Switzerland), bromodeoxyuridine (100 μmol/L; Merck, Buchs, Switzerland), vitamin C (18 μmol/L; Merck, Buchs, Switzerland), epinephrine (10 μmol/L; Merck, Buchs, Switzerland), L-glutamine (680 μM/L; Merck, Buchs, Switzerland), and 10% neonatal calf serum (NCS; Bioswisstech Ltd., Schaffhausen, Switzerland). The cell suspension underwent differential preplating in cell culture flasks for 120 min in order to separate CMCs from fibroblasts. Cell densities in the supernatant containing predominantly CMCs were determined with a hemocytometer and adjusted by dilution as to result in the required seeding density (5,000 cells/mm^2^). Cardiomyocyte cell cultures were kept in the incubator at 36°C in a humidified atmosphere containing 0.8% CO_2_. The culture medium was exchanged 24 h after seeding with supplemented medium M199 (cf. above) containing 5% NCS and every other day thereafter. At the time of the initial medium exchange, preparations were inspected by phase contrast microscopy for the presence of non-uniformities in cell density and the presence of holes. Preparations exhibiting these defects were excluded from further experiments. Experiments were performed 72 to 96 h after seeding of the CMCs.

### Patterned Growth Strand Preparations

Cardiomyocyte cell strands measuring 7.45 by 0.4 mm were produced using standard photolithography, with patterns being centered on the rows of electrodes on the multielectrode array (MEA) substrates (Rohr et al., 2003). A schematic illustration of the preparations is presented in Supplementary Figure S1.

### CMC-3T3_*HR*_ Cell Strands

3T3_*HR*_ cells were seeded at densities ranging from 250 to 2500 cells/mm^2^ onto the 24-h-old CMC cell strands. Cultures were incubated for 3 h to permit stable adherence of 3T3_*HR*_ cells to CMCs. Thereafter, the medium was replaced with fresh supplemented cell culture medium lacking bromodeoxyuridine. Preparations were kept in the incubator at 36°C in a humidified atmosphere containing 0.8% CO_2_ for a further 48 h before conducting the experiments.

### Determination of the Coverage Factor

The coverage factor (CF), that is, the relative cell membrane area of 3T3_*HR*_ cells in respect to the cell membrane area of CMCs (area 3T3_*HR*_ cells/area CMCs) of the preparations used in the MEA experiments, was determined by imaging the cell strands on an inverted microscope equipped for epifluorescence (Zeiss Axiovert 200M; excitation 482/35 nm; dichroic 499; emission 536/40 nm). Images were acquired with a cooled CCD camera (Spot RT-KAI2000; Spot Diagnostic Instruments, Sterling Heights, MI, United States). 3T3_*HR*_ cells were identified based on their eYFP fluorescence. With the objective used (2.5×, 0.075 N.A.), two images covered the entire length of the strands. They were stitched using Image Composite Editor (ICE; Microsoft, Redmond, WA, United States) and segmented with Ilastik (EMBL, Heidelberg, Germany), an interactive software that is trained to distinguish between fluorescent (foreground) and non-fluorescent areas (background). The segmented images were then processed using ImageJ (NIH, Bethesda, MD, United States), and the result, that is, the area covered by 3T3_*HR*_ cells, was divided by the area of the CMC strand. An example of a preparation is presented in Supplementary Figure S2.

### Conduction Measurements With Multielectrode Arrays

Impulse propagation characteristics in strands of CMCs (controls) and strands of CMCs coated with 3T3_*HR*_ cells were determined using a custom-built MEA system. The electrode layout of the MEA substrates was designed to our specification (EPFL, Neuchâtel, Switzerland) and is shown in Supplementary Figure S1. It consisted of four rows of recording electrodes that were regularly spaced (interelectrode distance: 0.5 mm) and terminated on either side by a stimulation dipole being positioned at 1 mm from the last recording electrode. Stimulation of the preparations and electrogram recordings were performed with a custom-built stimulation/recording system that provided an internal gain of 1,000. Stimulation dipoles and stimulation parameters (frequency, amplitude, duration) were software selectable. After digitization, data were transferred via a USB link to a PC that permitted following ongoing experiments in real time and was used for storing the data for later offline analysis using custom written software.

Experiments were performed in an incubator (36°C, 0.8% CO_2_) with preparations being stimulated by bipolar voltage pulses (±1 V; 4 ms) applied at 2.5 Hz to a stimulation electrode. After a prestimulation period of 50 s that allowed conduction to reach steady state, the recording was started, and the preparations subjected to a dark (20 s)–light (4 s)–dark (25 s) protocol. Preparations were illuminated by a high-brightness LED assembly (Luxeon Star 7 LEDs, SR-02-LO040; 590 nm, Lumileds, San Jose, CA, United States) equipped with a multilens collimating system (Cell Cluster Concentrator Optic # 263; Polymer Optics Ltd., Coventry, United Kingdom) that produced a spatially uniform light beam with a diameter of ∼10 mm that covered the entire preparation. Maximal light intensities reached at the level of the preparations amounted to 10.4 mW/mm^2^. To minimize warming of the preparation by the powerful light source, four fans removed the heat from the source. To ensure efficient heat removal, temperature was continuously monitored at the level of the MEA during the experiments.

Conduction velocities were assessed by software-based detection of local activation times defined by the minimum of the first derivative of the electrogram. From these data, conduction velocities were derived from the slope of a linear fit to the distance versus activation time plots.

### Whole-Cell Patch Clamp Recordings

For patch clamp experiments, low-density CMC and 3T3_*HR*_ cell cultures grown on glass coverslips were mounted in an experimental superfusion chamber (Rohr, 1986) and placed on the stage of an inverted microscope (Nikon Eclipse, TE2000-E, Nikon, Egg, Switzerland). Halorhodopsin was activated by a high-power LED (595 nm; Thorlabs, Thorlabs GmbH, Bergkirchen, Germany) that was coupled into the epi-illumination path of the microscope where it passed through an excitation filter (600/43 nm, Semrock, Rochester, NY, United States) followed by a dichroic mirror (570 nm, Semrock, Rochester, NY, United States). Light was projected onto the preparation by a 40×− 0.65 N.A. objective (Nikon). Maximal light power reached with this configuration at the level of the preparation was 8.9 mW/mm^2^. Preparations were superfused at 2 to 3 mL/min with HBSS containing 1% NCS at room temperature. Patch pipettes were pulled from borosilicate glass capillaries (GC150F-10, Harvard Apparatus, Holliston, MA, United States) with a horizontal puller (DMZ; Zeitz Instruments, Martinsried, Germany). The patch pipette filling solution contained (in mmol/L) K-aspartate 120, NaCl 10, Mg ATP 3, CaCl_2_ 1, EGTA 10, and HEPES 5 (pH 7.2; free Ca^2+^ concentration of ∼35 nmol/L). Pipette resistances ranged from 2 to 15 MΩ. Pipettes were positioned with a motorized micromanipulator (MP-225; Sutter Instrument Company, Novato, CA, United States). Pipette potentials were zeroed before cell contact, and measured potentials were corrected for the liquid junction potential (12.4 mV) as calculated by pCLAMP software (Molecular Devices LLC, San Jose, CA, United States). Analog signals were amplified, filtered (1 kHz), and digitized (2.9 kHz) with a HEKA EPC-10 patch clamp amplifier (HEKA Elektronik GmbH, Lambrecht, Germany). Data were stored on a computer for offline analysis with Patchmaster (V 2.15) and Fitmaster (V 2.53) software (HEKA).

Electrophysiological properties of 3T3_*HR*_ cells were assessed using standard whole-cell patch clamp recording techniques. Seal resistances ranged from 2 to 10 GΩ. After rupture of the membrane, series resistance, membrane capacitance, and the liquid junction potential were compensated. Membrane resistances and cell capacitances were calculated by Patchmaster. Current-to-voltage (I–V) relationships were obtained by applying continuous voltage ramp protocols where, starting from a holding potential of −70 mV, the membrane was clamped to −100 and ramped to 50 mV within 1.2 s (slew rate of 125 mV s^–1^). In case of 3T3_*HR*_ cells, ramps were performed first in the dark and then in presence of HR-activating light (590 nm). The light intensity used in the experiments (8.9 mW/mm^2)^ has been shown before to cause maximal activation of the optogenetic actuator eNpHR3.0-eYFP (Zhang et al., 2019). In agreement with these data, we found previously in eNpHR3.0-eYFP–transduced cardiac myofibroblasts that photocurrents saturate at light intensities ≥3.5 mW/mm^2^. Net whole-cell currents were normalized to cell capacitance and are reported as pA/pF. The magnitude of the HR-dependent chloride pump current and its reversal potential were calculated from the difference between the I–V curves measured in the presence of light and in the dark. The time course and size of light-induced hyperpolarizations was measured in current clamp mode (I = 0). Maximal light-induced voltage changes were determined 0.5 s after the start of illumination, which corresponds to ∼5 × the activation time constant (cf. Supplementary Figure S4).

### Computer Simulations of Conduction

Monodomain computer simulations of action potential propagation were conducted in a fiber containing 100 CMCs, each having a length of 60 μm and a capacitance C_*CMC*_ of 28.8 pF. Ion currents of CMCs were based on a model that was designed to reproduce findings in strands of neonatal rat ventricular myocytes cocultured with myofibroblasts (Jousset et al., 2016). The lumped gap junctional and myoplasmic conductance between CMCs was adjusted to 0.38 μS to replicate the mean control θ measured in the present study in the absence of 3T3_*HR*_ cells.

Coating of CMC cell strands with 3T3_*HR*_ cells at different CFs was simulated by connecting each CMC laterally to a 3T3_*HR*_ cell having a capacitance C_3__*T*__3__*HR*_ = CF ⋅ C_*CMC*_. Ion currents of the 3T3_*HR*_ cells (*I*_*dark*_: total dark current; *I*_*HR*_: HR-induced light-activated current) were modeled based on current–voltage relationships (normalized to cell capacitance) as obtained in patch clamp experiments. Dark conditions were simulated by setting *I*_*HR*_ to 0. The absolute membrane current of 3T3_*HR*_ cells was obtained by multiplying it by C_3__*T*__3__*HR*_. In an additional set of simulations, both *I*_*dark*_ and *I*_*HR*_ were set to 0, which resulted in purely capacitive cells having the same RMP as CMCs.

3T3_*HR*_ cells were connected to CMCs by a conductance g_*CMC–*__3__*T*__3__*HR*_ of 72 nS and scaled by the CF. Scaling both C_3__*T*__3__*HR*_ and g_*CMC–*__3__*T*__3__*HR*_ by the CF ensured that the time constant determining the passive loading of 3T3_*HR*_ cells (C_3__*T*__3__*HR*_/g_*CMC–T*__3__*HR*_) remained invariant at 0.4 ms, a value based on previous experimental observations in our laboratory (Salvarani et al., 2017). In additional simulations, the effects of a decreased CMC-3T3_*HR*_ coupling (1.2 nS) on conduction were investigated. 3T3_*HR*_ cells were not connected to each other.

Different CFs were modeled by scaling the capacitance of the 3T3_*HR*_ cells, their ionic currents, and their intercellular coupling conductance to the CMCs. This approach represents a homogenization of the distribution of the 3T3_*HR*_ cells. In a previous study using a detailed cellular structure model, we observed that a random distribution of myofibroblasts on CMCs leads to only minimal spatial variations of the RMP (<0.1 mV) and to equally spaced isochrones at a macroscopic level (Jousset et al., 2016). Thus, the homogenized model is adequate for characterizing macroscopic θ.

Simulations were started with a period of no stimulation (2 s) to allow the CMCs and the 3T3_*HR*_ cells to reach their respective RMPs and to ascertain the absence of spontaneous activity. The fiber was then stimulated by a rectangular current pulse applied to the first CMC. The intensity and duration of the stimulus were adjusted in each simulation to ensure that a propagated action potential was properly initiated without a major stimulation artifact.

Activation time was defined as the time at which the membrane potential of CMCs surpassed –35 mV during depolarization. Conduction velocity was computed by linear regression of activation time versus distance between cell 25 and cell 75 to exclude stimulation artifacts and sealed-end effects. In the CMC model, the Na^+^ current (*I*_*Na*_) was represented using a Hodgkin–Huxley formalism with three activation gates (m) and two inactivation gates (h and j) (Luo and Rudy, 1991; Jousset et al., 2016). The availability of *I*_*Na*_ was calculated as the product of the steady-state values of h and j at the RMP. Extracellular potentials were reconstructed at a distance of 5 μm from the 50th cell of the fiber using the method of Plonsey and Barr (Plonsey and Barr, 2000) and assuming an extracellular resistivity of 100 Ω cm.

The system of differential equations was integrated numerically using a constant time step of 0.005 ms. Gating variables were integrated using the method of Rush and Larsen (1978) and membrane potentials were determined using the forward Euler method. All simulations were run using MATLAB (The MathWorks, Natick, MA, United States).

### Statistical Analysis

Normal distribution of data was assessed using the Shapiro–Wilk test. Data comparison was performed with the Student *t*-test (homoscedastic or heteroscedastic where appropriate). Differences were considered significant at *p* < 0.05.

## Results

### Electrophysiological Characterization of 3T3_*HR*_ Cells

**FIGURE 1:**
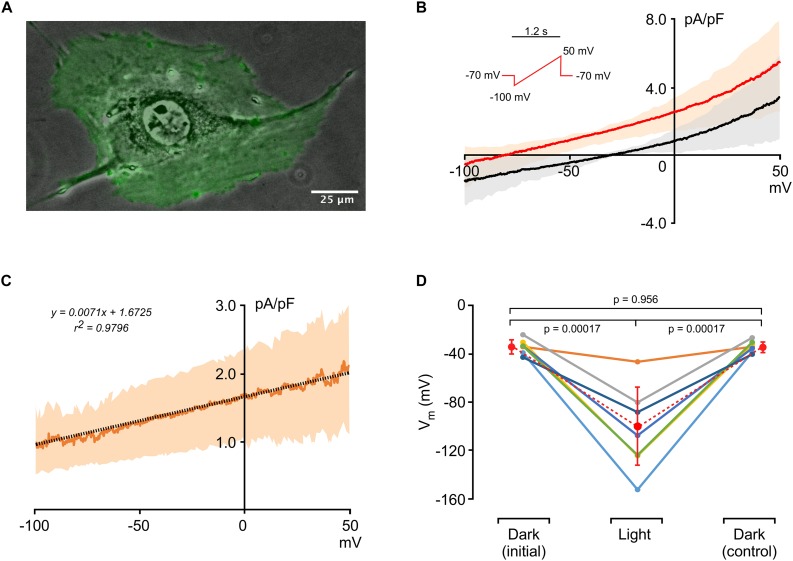
Electrophysiological characterization of 3T3_*HR*_ cells. **(A)** Fluorescence image of an HR-expressing 3T3 cell (green) overlaid on a phase contrast image. **(B)** Current-to-voltage relationships of 3T3_*HR*_ cells in the dark (black) and during activation of HR (red) (*n* = 8; mean ± SD; SD spread indicated by a light-red and gray band; voltage clamp protocol shown in the inset). **(C)** Difference current (*I*_*light*_ − *I*_*dark*_) induced by activation of HR at saturating light intensities (590 nm; 8.9 mW/mm^2;^ *n* = 8; mean ± SD). The linear fit (stippled black line) indicates that the actuator-induced outward current reverses at ∼−235 mV. **(D)** Membrane potentials (*V*_*m*_) of single 3T3_*HR*_ cells before, during, and after HR activation (*n* = 7: 590 nm; 8.9 mW/mm^2^).

An image of an HR-expressing single 3T3 cell (3T3_*HR*_) is shown in Figure 1A. The optogenetic actuator fused to eYFP is uniformly expressed as evidenced by the homogeneous green fluorescence. Currents recorded during application of voltage ramps (-100 to 50 mV within 1.2 s) are shown in Figure 1B and indicate slight outward rectification of currents at positive potentials. Membrane potentials of 3T3_*HR*_ cells in absence of HR activation ranged from -26 to -44 mV (average, 35.0 ± 5.4 mV; *n* = 7), membrane capacitances from 43 to 78 pF mV (average, 64.7 ± 11.3, *n* = 7), and input resistances from 0.5 to 1.4 GΩ (0.95 ± 0.33 GΩ, *n* = 7). During HR activation at saturating light intensities (590 nm; 8.9 mW/mm^2^), the current-to-voltage (I–V) curve was shifted upward due to the light-induced Cl^–^ pump current produced by HR. A linear fit applied to the HR-dependent current indicated a reversal potential at approximately −235 mV (Figure 1C). In current clamp mode with I = 0, activation of HR caused a significant hyperpolarization of the membrane potential of 3T3_*HR*_ cells from −35.0 ± 5.4 mV (dark conditions) to −103.9 ± 31.9 mV (illuminated; *n* = 7; *p* = 0.0017), with average membrane potentials before and after HR activation (−35.0 ± 5.4 mV vs. −35.0 ± 4.4 mV) being identical (Figure 1D). As shown in Supplementary Figure S3, the magnitude of *I*_*HR*_ showed a close to linear dependence on the level of expression of the optogenetic reporter, which ranged from 3.0 to 33.3 (a.u.) with *I*_*HR*_ measured at a clamp potential of 0 mV increasing from 0.53 pA/pF to 2.58 pA/pF (linear fit: slope 0.06, *r*^2^ = 0.81, *n* = 5). Time constants of the light-induced hyperpolarizing responses were 94.3 ± 46.4 ms (on-response; *n* = 18) and 137.7 ± 58.9 ms (off-response; *n* = 15; Supplementary Figure S4). Supplementary Figure S5 depicts an example of a 3T3_*HR*_ cell–CMC cell pair with the contact region showing discrete immunofluorescence staining for connexin43.

### HR Activation Only Partially Restores θ in 3T3_*HR*_–CMC Strand Preparations

**FIGURE 2:**
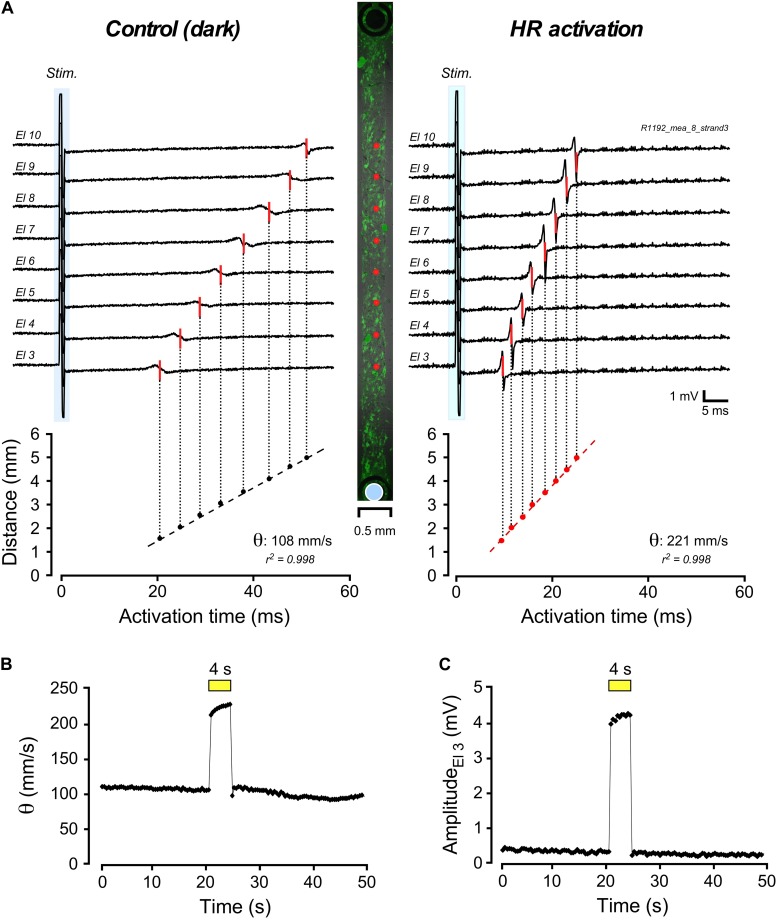
Multielectrode array–based assessment of conduction in heterologous CMC-3T3_*HR*_ cell strands. **(A)** Electrograms measured along a 400-μm-wide strand of CMCs coated with 3T3_*HR*_ cells (center panel: fluorescence image of 3T3_*HR*_ cells with red dots indicating recording sites and the blue disk denoting the stimulation dipole). Compared to the control recordings in the dark (left panel; stimulation artifact marked with blue backdrop), electrical activation of the preparation was accelerated during HR stimulation (right panel). **(B)** Time course of the increase of conduction velocity (θ) during transient light stimulation of HR (yellow bar; 590 nm; 10.4 mW/mm^2^). **(C)** Time course of the change of electrogram amplitude as recorded by electrode #3 during light stimulation of HR (yellow bar, same experiment).

Impulse conduction along strands of CMCs coated at increasing densities with 3T3_*HR*_ cells was assessed using a MEA system. Conduction velocities (θ) in strands of CMCs were not significantly altered by illumination (dark: 333 ± 59 mm/s; light-on: 338 ± 54 mm/s; *n* = 25). Also, electrogram amplitudes (1.5 ± 0.8 mV; *n* = 25) and downstroke times (time from the maximum to the minimum of the electrogram; 0.29 ± 0.04 ms; *n* = 25) remained unchanged. By contrast, CMC strands coated with 3T3_*HR*_ cells showed a distinct response to HR activation as is illustrated in Figure 2 for a preparation with a CF of 0.65. The center panel of Figure 2A depicts the preparation with green fluorescent 3T3_*HR*_ cells and the positioning of the stimulation (blue disk) and recording electrodes (red dots). Impulse conduction under control, that is, dark conditions, was uniform and, as expected for heterocellular preparations, slow (108 mm/s; left panel). Uniform illumination of the cell strand (590 nm; 10.4 mW/mm^2^) caused, as shown in the right-hand panel, a substantial acceleration of θ to 221 mm/s. The temporal profile of the light-induced change in impulse conduction is shown in Figure 2B. θ responded to illumination with an instantaneous increase by over 100% and promptly returned to preillumination values after switching off the light. Apart from accelerating conduction, activation of HR was accompanied by a several-fold increase of the electrogram amplitude as exemplified by the electrogram of electrode #3 in Figure 2C. When averaging all electrograms (*n* = 8) of this preparation, amplitudes increased ∼4.4-fold from 0.66 ± 0.13 mV in the dark to 2.88 ± 0.49 mV during HR activation (*p* < 2.3 × 10^–6^).

### Partial Restoration of Conduction Velocity During HR Activation Depends on 3T3_*HR*_ Cell Density

**FIGURE 3:**
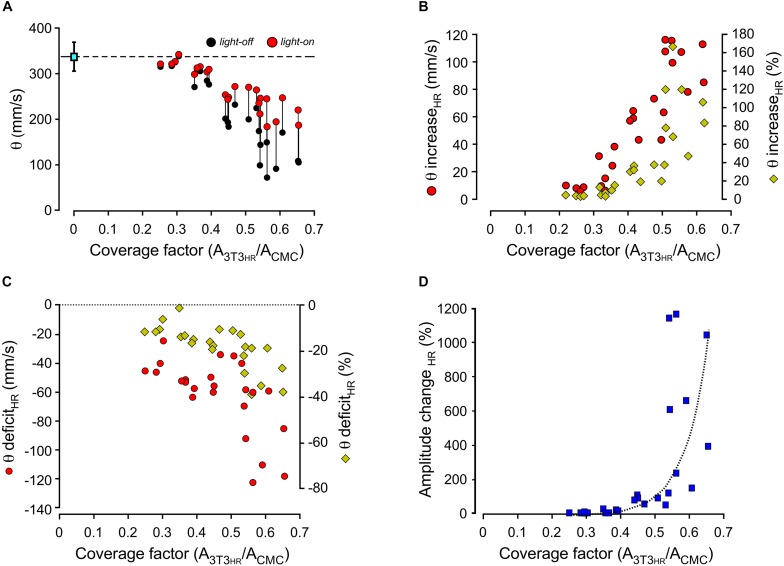
Dependence of conduction velocities and electrogram amplitudes on the coating density of 3T3_*HR*_ cells. **(A)** Conduction velocities as a function of the CF in the dark (black dots) and during HR activation (red dots; 590 nm; 10.4 mW/mm^2^). **(B)** Increase of conduction velocities in CMC-3T3_*HR*_ cell strands in response to illumination (left ordinate: red symbols, absolute increase in mm/s; right ordinate: yellow diamonds, relative increase in respect to conduction velocities measured in the dark). **(C)** Persistent deficit of conduction velocities during HR activation in CMC-3T3_*HR*_ cell strands as compared to control CMC strands (left ordinate: red symbols, persistent conduction slowing in absolute values; right ordinate: yellow diamonds, persistent conduction slowing relative to CMC control strands; the stippled line refers to conduction velocities measured in CMC control strands). **(D)** Relative increase of electrogram amplitudes in response to HR activation plotted as a function of 3T3_*HR*_ cell density.

Compared to conduction velocities measured in CMC preparations without 3T3_*HR*_ cells (333 ± 6 mm/s; *n* = 25), strands coated with 3T3_*HR*_ cells exhibited slow conduction. The degree of conduction slowing was correlated to 3T3_*HR*_ cell density, and values as low as 100 mm/s were observed at the largest CFs used (Figure 3A, black symbols). When removing the depolarizing effect of 3T3_*HR*_ cells on coupled CMCs by activating HR, θ consistently increased for CFs > 0.3 (Figure 3A, red symbols). As shown in Figure 3B, the increase of θ was correlated to the density of 3T3_*HR*_ cells in both absolute and relative terms. At high 3T3_*HR*_ cell coverage, the light-induced increase of θ relative to initial values in the dark was as high as 160%. Despite this substantial recovery of conduction during light activation of HR, there remained a θ deficit in respect to CMC cell strands that was positively correlated to 3T3_*HR*_ cell density (Figure 3C). At the highest 3T3_*HR*_ cell densities tested, θ remained depressed by ∼40% (as opposed to ∼70% in absence of HR stimulation), which, as demonstrated by the simulations presented in the following section, reflects capacitive loading of the CMCs by electrotonically coupled 3T3_*HR*_ cells. Finally, *I*_*HR*_ activation by light not only accelerated conduction but, due to the removal of partial inactivation of the sodium current in coupled CMCs, caused a CF-dependent increase in peak-to-peak amplitudes of the electrograms that increased several-fold at large CFs compared to peak-to-peak amplitudes in the dark (Figure 3D).

### *In silico* Assessment of Conduction in Heterologous CMC-3T3_*HR*_ Cell Strands

The plausibility of the argument that activation of HR exposes the capacitive load (CL) of 3T3_*HR*_ cells exerted on coupled CMCs by removing their depolarizing effect, that is, their resistive load (RL) component, was evaluated by comparing experimental findings to *in silico* models of conduction along strands of CMCs coupled to non-excitable 3T3_*HR*_ cells.

**FIGURE 4:**
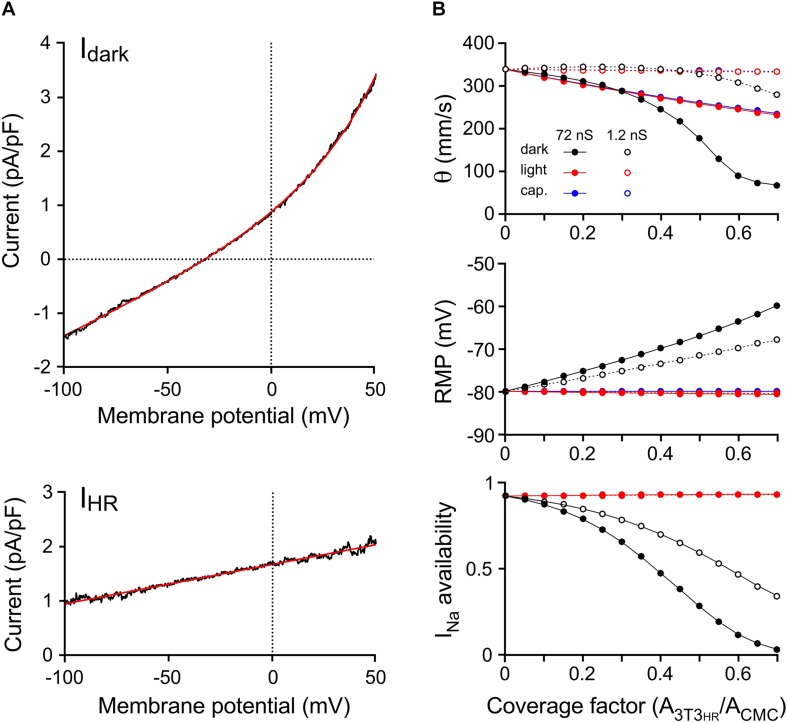
Computer simulations of 3T3_*HR*_ cell electrophysiology and impulse conduction. **(A)** Mean current-to-voltage relationships of 3T3_*HR*_ cells during voltage clamp ramps in the absence of light (*I*_*dark*_, top) and during HR activation with light (net light induced HR pump current, *I*_*HR*_, bottom). The black curves denote mean values (*n* = 8), whereas the red curves represent the fitted functions used in the 3T3_*HR*_ cell model [*I*_*dark*_ = 0.46862⋅e^*V/*33.751^ + 0.018636⋅V + 0.42087; *I*_*HR*_ = 0.0072742 ⋅ (*V* + 229.95)]. **(B)** Conduction velocity (top), RMP (middle), and *I*_*Na*_ availability (bottom) versus CF for CMC-3T3_*HR*_ cell strands exhibiting normal (72 ns = 720 connexons; solid lines) and reduced gap junctional coupling (1.2 ns = 12 connexons; stippled lines). Simulations refer to dark conditions (black), light stimulation (red), and to the case of purely capacitive 3T3_*HR*_ cells (blue). At CFs > 0.7, spontaneous activity occurred in the dark, and θ at a stable RMP could not be determined anymore.

Ion currents present in CMCs were based on our previously developed model (Jousset et al., 2016). In simulated CMC control strands, intercellular coupling was adjusted as to replicate control conduction velocities observed in CMC strand preparations devoid of 3T3_*HR*_ cells (experimentally observed velocities: 333 ± 6 mm/s; *n* = 25; lumped myoplasmic and junctional conductance between myocytes in the model of 0.3817 μS yielded an overall θ of 339 mm/s). The ion currents of 3T3_*HR*_ cells were modeled as a function of the membrane potential based on the patch clamp experiments presented in Figure 1. Figure 4A shows the mean total dark current density (*I*_*dark*_, black) and the mean HR-mediated current density (*I*_*HR*_, black) induced by illumination, together with the fitted functions used to represent these currents in the model (red).

The effects of increasing densities of 3T3_*HR*_ cells on θ and the RMP of CMCs are shown in Figure 4B. Simulations were run in the absence of light (black symbols), in the presence of the light-induced *I*_*HR*_ (red symbols), and for two different levels of heterocellular gap junctional coupling. The coupling conductance of 72 nS (filled symbols) was based on values found before in pairs of CMCs coupled to non-excitable myofibroblasts (Salvarani et al., 2017). Additionally, we performed simulations at very low levels of intercellular coupling [open symbols; 1.2 nS corresponding to ∼12 connexons when assuming a single connexon conductance of 100 pS (Valiunas et al., 1997)].

At normal levels of CMC-3T3_*HR*_ coupling and in absence of *I*_*HR*_, increasing the CF led to a prominent conduction slowing (339 mm/s to 67 mm/s) because the moderately polarized 3T3_*HR*_ cells (*V*_*m*_ of the isolated model 3T3_*HR*_ cell: −32.3 mV) induced a reduction of the RMP of CMCs from −79.9 mV to −59.9 mV. This depolarization caused partial inactivation of the sodium channels as quantified by *I*_*Na*_ availability (bottom panel of Figure 4B). For CFs > 0.6, *I*_*Na*_ was almost fully inactivated, and slow conduction was essentially supported by the L-type Ca^2+^ current, which is in agreement with previous findings with CMC strands being depolarized by a rise in [K^+^]_*o*_ or CMC strands coated with myofibroblasts (Rohr et al., 1998; Miragoli et al., 2006). During HR activation, the depolarizing effect of 3T3_*HR*_ cells on coupled CMCs was entirely suppressed, *I*_*Na*_ availability fully restored, and conduction accelerated for CFs > 0.3. Importantly, and in agreement with the experiments (Figure 3), θ did not fully return to levels observed in control CMC strands indicating that CMC depolarization, that is, resistive loading, was not the only determinant of 3T3_*HR*_ cell–induced conduction slowing.

To investigate whether the reduction of θ persisting in the presence of *I*_*HR*_ was due to the CL exerted by the 3T3_*HR*_ cells on coupled CMCs, both *I*_*dark*_ and *I*_*HR*_ of 3T3_*HR*_ cells were set to 0, resulting in 3T3_*HR*_ cells that acted in a purely capacitive manner (Figure 4B, blue symbols). While this intervention did not affect the RMP of coupled CMCs, it led to a slowing of conduction that was virtually identical to that observed in the presence of both *I*_*dark*_ and *I*_*HR*_. This finding supports the conclusion that the incomplete recovery of θ observed in CMC-3T3_*HR*_ cell preparations during activation of *I*_*HR*_ was primarily caused by the CL imposed by the 3T3_*HR*_ cells on the coupled CMCs.

At low levels of CMC-3T3_*HR*_ cell coupling (Figure 4B, open symbols), increasing the density of 3T3_*HR*_ cells first led to a slight increase and then a moderate decrease of conduction velocities to 280 mm/s, which was paralleled by a steady decrease of the RMP of CMCs from −79.9 to −68.0 mV and a reduction of *I*_*Na*_ availability to 36.5%. The phenomenon of a transient increase of θ at CFs < 0.3 is reminiscent of supernormal conduction and is explained by the fact that the depolarization caused by the 3T3_*HR*_ cells brought the RMP of CMCs closer to the threshold of *I*_*Na*_ activation. Because *I*_*Na*_ inactivation at these low CFs was still moderate, the reduction of the source current necessary to activate *I*_*Na*_ prevailed, and hence, θ increased at CF < 0.3.

For the case of weak coupling of 3T3_*HR*_ cells to CMCs, θ returned to control values during HR activation. This is in contrast to the experimental observations and suggests that the coupling conductance between CMCs and 3T3_*HR*_ cells in the experimental preparations was closer to 72 nS than 1.2 nS and that robust heterocellular coupling is prerequisite for 3T3_*HR*_ cells to exert a significant CL on neighboring CMCs.

### 3T3_*HR*_ Cell–Induced Electrogram Amplitude Changes: Simulation Versus Experiment

**FIGURE 5:**
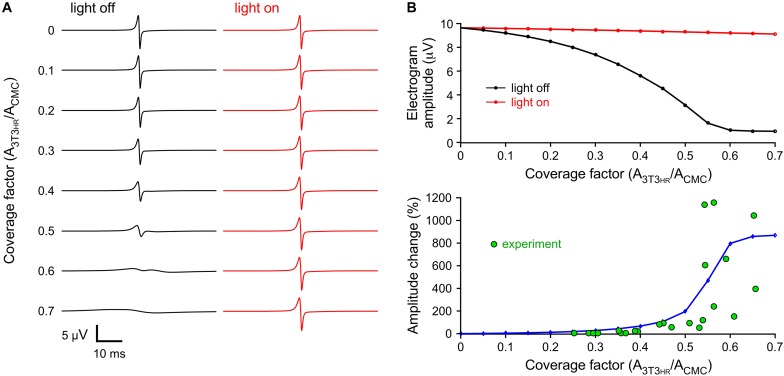
Simulated extracellular electrograms. **(A)** Electrograms under dark conditions (light off, black) and during light stimulation (light on, red) for increasing CFs and a CMC-3T3_*HR*_ cell coupling conductance of 72 ns. **(B)** Corresponding electrogram amplitudes (top) and relative amplitude changes upon illumination (blue, bottom) versus CF. The green symbols represent the values observed in the experiments.

3T3_*HR*_ cell density-dependent changes in peak-to-peak electrogram amplitude were investigated by computing electrograms at a distance of 5 μm from the middle of the strand (CMC-3T3_*HR*_ cell coupling conductance of 72 nS). As shown in Figure 5A, electrogram amplitudes decreased with increasing 3T3_*HR*_ cell densities from 9.6 μV (control) to 0.94 μV at a CF of 0.7. This decrease was nearly fully reversed upon HR activation. Specifically, at the highest 3T3_*HR*_ cell densities, electrogram amplitudes were restored by more than 94% as compared to control conditions (Figure 5B, upper panel). The computed relative changes in electrogram amplitudes upon *I*_*HR*_ activation (Figure 5B, lower panel) were in qualitative agreement with the experimental values, indicating that the parameters chosen for the cell models were adequate. For simulations using a very low coupling conductance (1.2 nS), the effects of resistive loading by coupled 3T3_*HR*_ cell were, as expected, reduced (9.7 μV to 4.7 μV; Supplementary Figure S6).

### 3T3_*HR*_ Cell–Induced Conduction Slowing: Simulation Versus Experiment

**FIGURE 6:**
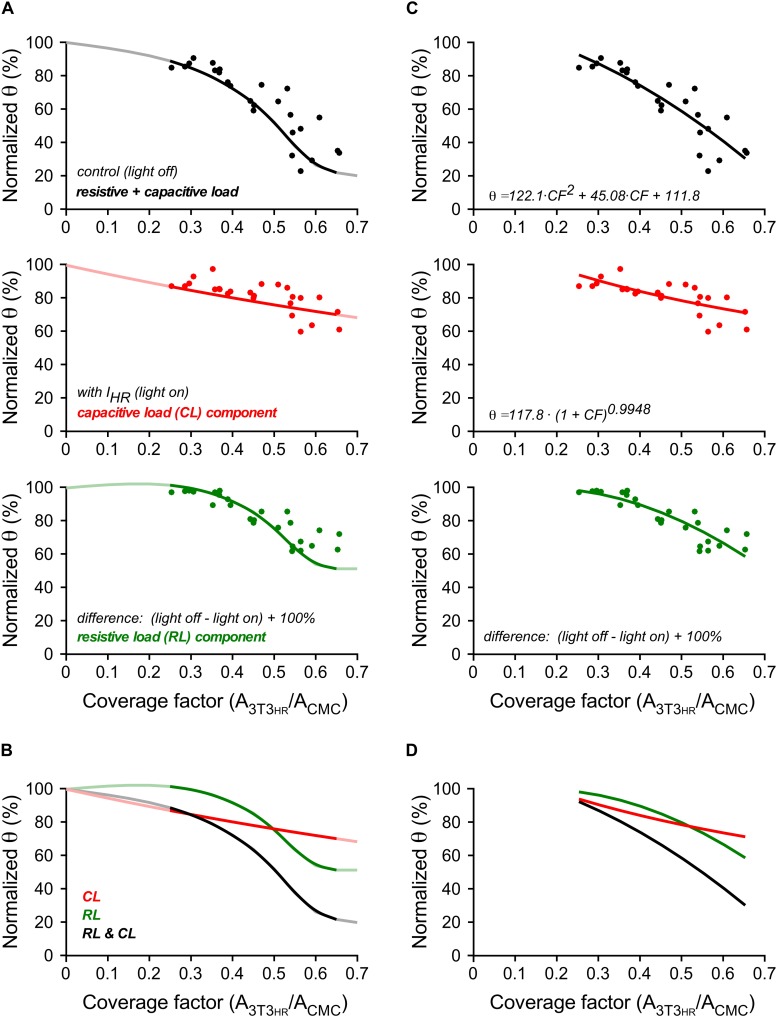
Conduction velocities in 3T3_*HR*_-coated CMC cell strands in experiments and computer simulations. **(A)** Normalized conduction velocities versus CF in experiments (dots) versus simulations (curves). Top panel: light-off conditions (black); middle panel: light-on conditions (red); bottom panel: difference between light-off and light-on conditions corresponding to the effect of resistive loading on θ (green). **(B)** Overlay of the curves shown in panel **(A)**. **(C)** Normalized θ versus CF in experiments (dots, same data and layout as in panel **A**). Black curve: quadratic fit. Red curve: power function fit. Green curve: computed as in panels **A,B** from the fitted functions. **(D)** Overlay of the curves shown in panel **(C)**.

A direct comparison of experimental and simulated conduction velocities for different 3T3_*HR*_ cell densities is shown in Figure 6A. Within the range of CFs present in experiments (0.25 < CF < 0.65), simulation (solid curves) and experiments (single symbols) showed a high degree of overlap. As hypothesized, the θ deficit persisting during activation of *I*_*HR*_ (light-on conditions; red symbols) was closely matched by the simulation data describing capacitive loading (red line). The effects of resistive loading (green symbols) were obtained by subtracting the normalized velocities obtained during *I*_*HR*_ activation from the normalized velocities in the dark and adding an offset of 100% to the results. Again, the results closely matched the simulation data of the effect of resistive loading on impulse propagation (green line). The overlay of all signals shown in Figure 6B illustrates that resistive loading (sigmoidal) and capacitive loading (monotonic) are equally effective in slowing conduction at a CF of ∼0.5. At lower 3T3_*HR*_ cell densities, capacitive loading prevails over resistive loading in slowing conduction and vice versa at CFs > 0.5.

Figures 6C,D illustrate an additional quantification based on curve fitting. Experimental values obtained in the dark were fitted with a polynomial function (*r*^2^ = 0.78). The data obtained during illumination representing capacitive loading were fitted by a power function (*r*^2^ = 0.51) based on theoretical considerations by Huxley (Huxley, 1959). The power function related θ to the total membrane capacitance present, that is, 1 (for the CMCs) + CF (for the 3T3_*HR*_ cells). From the fitted functions, the expected effect of resistive loading (CMC depolarization) was calculated as described above. Besides confirming that the effect of *I*_*HR*_ on θ is larger for higher 3T3_*HR*_ cell densities, the analysis permitted the estimation of the power law exponent describing the effect of membrane capacitance on θ. For the experimental data presented, a value of −0.99 was obtained (95% confidence interval = −1.42 to −0.57). For the simulations (red curve in Figure 6A), the same fitting procedure resulted in an exponent of −0.71 (95% confidence interval = −0.73 to −0.68).

## Discussion

The present study was designed with the goal to experimentally determine the relative contributions of resistive versus capacitive loading to conduction slowing as induced in cardiac tissue by electrotonically coupled NECs (Miragoli et al., 2006; Kucera et al., 2017). The results demonstrate that capacitive loading of CMCs by coupled NECs is similarly important for conduction slowing as resistive loading.

### Membrane Capacitance and Impulse Propagation: Theoretical Aspects

From a theoretical point of view, it is known for many decades that the membrane capacitance of excitable cells affects propagation. Quantitatively, this problem was addressed by Hodgkin in 1959, as reviewed in Jack et al. (1983). For a homogeneous, infinitely long, and uniform excitable structure (such as a non-myelinated axon), the inverse square law relating θ and axial resistance can be easily derived from cable theory. However, for the same uniform excitable structure, the relation between θ and membrane capacitance cannot be derived analytically. The problem arises from the kinetic behavior, that is, the time dependence of the ion currents underlying the action potential. While membrane capacitance directly influences the speed at which the membrane is charged (and discharged) by ion currents, the time-dependent gating of currents (mainly of *I*_*Na*_, the primary ion current determining θ) exerts a feedback on the rate of rise of the action potential upstroke. Nevertheless, based on the theoretical considerations of Hodgkin, the relation between θ and capacitance is expected to follow a power law behavior with an exponent between −1/2 and −1, depending on the nature of the ion currents. However, the exact exponent can only be determined empirically for a given excitable preparation or mathematical model.

### Membrane Capacitance and Impulse Propagation: Existing Experimental Evidence

Membrane capacitance dependent changes in impulse propagation are expected to occur when the membrane area of conducting structures increases while, simultaneously, the total amount of transmembrane currents available to charge the cells to the activation threshold remains unchanged. Such a mechanism has been proposed to underlie, for example, stretch-dependent slowing of conduction in cultured mouse CMCs with unfolding of caveolae being the source of the increased membrane area (Pfeiffer et al., 2014). The same situation is present when NECs or cells exhibiting a reduced excitability compared to CMCs are electrotonically coupled to the latter by either gap junctions or nanotubular structures (Davis and Sowinski, 2008; Quinn et al., 2016). Non-excitable cells that have been shown to establish heterocellular electrotonic coupling to CMCs include cardiac myofibroblasts (Miragoli et al., 2006; Quinn et al., 2016; Salvarani et al., 2017), HeLa cells expressing connexin43 (Gaudesius et al., 2003), macrophages (Hulsmans et al., 2017), and NIH3T3 cells (Haraguchi et al., 2010; Nussinovitch et al., 2014). Because these cell types commonly exhibit a reduced membrane potential compared to CMCs, their net effect on impulse propagation is determined not only by representing an additional capacitance (CL) but also by their depolarizing effect on coupled CMCs (RL). To complicate matters further, the two effects are not simply additive because, in contrast to the theoretically expected gradual slowing of conduction with increasing membrane capacitance, increasing levels of membrane depolarization of CMCs by coupled NECs will result in a biphasic change of conduction due to the phenomenon of supernormal conduction (Kagiyama et al., 1982; Shaw and Rudy, 1997; Rohr et al., 1998; Jacquemet and Henriquez, 2008).

### Untangling the Relative Contributions of Resistive and Capacitive Loading to Conduction

In the past, experimental characterizations of the effects of NECs coupled to CMCs on impulse propagation were necessarily limited to descriptions of the combined effect of both resistive and capacitive loading because experimental techniques suitable to differentiate between RL and CL were missing. Ideally, deciphering the individual contributions of RL and CL would consist of reversibly annihilating either the resistive or the capacitive component. Whereas the latter approach is inconceivable, removing the depolarizing effect of NECs on coupled CMCs can be achieved by letting NECs express an optogenetic membrane voltage actuator such as eNpHR3.0 (Gradinaru et al., 2010) that hyperpolarizes NECs during illumination into the range of RMPs of CMCs, thereby removing the RL component and, consequently, unmasking the role of capacitive loading in impulse conduction slowing.

### Choice of Experimental NEC Model

Even though the effects of coupled NECs on cardiac impulse propagation have been extensively characterized in the past with primary cardiac myofibroblasts (Gaudesius et al., 2003; Miragoli et al., 2006; Rosker et al., 2011; Salvarani et al., 2017), we resorted to NIH3T3 cells as a model for NECs in this study because pilot experiments had shown that a uniform transduction of cardiac myofibroblasts with eYFP- eNpHR3.0 was difficult to achieve. By contrast, NIH3T3 cells permitted to establish a cell line that stably expressed eYFP-eNpHR3.0 (3T3_*HR*_ cells). This ascertained that light activation of HR involved all NECs present in the heterologous cell strands, which was a prerequisite for attributing observed effects of HR activation in full to the NECs. As shown in this study, 3T3_*HR*_ cells exhibited I–V relationships and membrane potentials (∼−35 mV) similar to that of myofibroblasts [∼−27 mV (Salvarani et al., 2017)]. Also, 3T3_*HR*_ cells reduced θ in heterologous strand preparations by more than 60% at the highest seeding densities used, which matches previous findings with cardiac myofibroblasts (Miragoli et al., 2006). Finally, the small electrogram amplitudes found in slowly conducting preparations indicated that slow conduction, similar to myofibroblasts, was dependent on sodium current inactivation secondary to 3T3_*HR*_ cell–induced CMC depolarization, that is, resistive loading. Overall, these findings suggested that primary cardiac myofibroblasts can be substituted by 3T3_*HR*_ cells when probing the relative contributions of CL and RL to conduction slowing in coupled CMCs.

Whereas in the study by Miragoli et al. (2006) myofibroblast density was given as cell count per area, we used a different measure in this study, that is, the CF (CF: area covered by 3T3_*HR*_ cell divided by area covered by CMCs). The rationale of using this measure relates to the fact that it is ultimately the relative size of the membrane areas of the two cell types and not the cell count that determines the extent of resistive and capacitive loading.

### Light Stimulation of 3T3_*HR*_ Cells: Effects at the Single-Cell Level and in Multicellular Preparations

Light activation of single 3T3_*HR*_ cells caused their *V*_*m*_ to shift from ∼−35 to ∼−104 mV. While this degree of hyperpolarization eliminates resistive loading of CMCs by coupled 3T3_*HR*_ cells, the question arises whether light activation may in fact lead to a hyperpolarization of CMCs relevant for conduction (Funken et al., 2019). As reported before (Salvarani et al., 2017), CMC membrane potential changes induced by coupled NECs depend on the relative sizes of the membrane resistance of NECs (∼950 MΩ), the membrane resistance of CMCs [∼290 MΩ, from Salvarani et al. (2017)], and the gap junctional resistance [14 MΩ, from Salvarani et al. (2017)]. Given these resistances, light-activated 3T3_*HR*_ cells (*V*_*m*_ of −104 mV) would hyperpolarize coupled CMC (RMP of −80 mV) by ∼5 mV for a CF of 1 and by maximally 3 mV for the highest CFs used in this study (0.7), which is in the same range as in the simulations (∼1 mV at a CF of 0.7) and excludes a relevant effect of 3T3_*HR*_ cells being hyperpolarized by light beyond the RMP of CMCs on conduction.

Illumination of strand preparation coated with 3T3_*HR*_ cells caused a CF-dependent increase of electrogram amplitudes and an acceleration of impulse conduction. At the highest 3T3_*HR*_:CMC ratios used, electrogram amplitudes increased ∼10-fold, and θ increased by up to 120 mm/s (absolute) or up to 2.8-fold (relative). Both of these effects are consistent with the concept that light stimulation of 3T3_*HR*_ cells caused normalization of the RMP of coupled CMCs and hence lead to a recovery of sodium current availability. As hypothesized, this illumination-induced annihilation of resistive loading of CMCs by coupled 3T3_*HR*_ cells was not sufficient to fully restore θ to values measured in control CMC cell strands. The respective deficit, which likely represented the contribution of capacitive loading to impulse conduction slowing, amounted to ∼30% at the highest 3T3_*HR*_ CFs investigated.

### Effects of Resistive and Capacitive Loading on Conduction: Experiment and Computer Simulation

Because there exists no experimental approach to prove directly that the residual conduction slowing observed during light stimulation of 3T3_*HR*_ cells reflects the capacitive load component exerted by NECs on coupled CMCs, we tested this hypothesis in computer simulations of fibers of CMCs only and fibers of CMCs coated at increasing densities with 3T3_*HR*_ cells. The model was based on a previously developed CMC model (Jousset et al., 2016) and a model of 3T3_*HR*_ cells specifically developed for this study that was based on our patch clamp data. Simulated 3T3_*HR*_ cells were coupled to CMCs with a conductance of 72 nS as determined before in myofibroblast–CMC cell pairs (Salvarani et al., 2017) and, for comparison purposes, at a reduced coupling conductance of 1.2 nS. The latter assumption of heterocellular coupling strength was inadequate because, unlike experimental findings, θ was largely unaffected at CFs up to 0.5, and at larger CFs, activation of 3T3_*HR*_ cells led to an almost full recovery of conduction. By contrast, results from simulations using a heterocellular coupling conductance of 72 nS closely reflected the experimental findings.

Starting from control conduction velocities as determined in CMC strands, non-stimulated 3T3_*HR*_ cells reduced θ in a CF-dependent manner from ∼340 mm/s to less than 100 mm/s, thereby closely mimicking experimental observations made before with myofibroblasts. The CF-dependent decrease of θ showed multiple phases with a slow initial decline (CF < 0.3) being followed by a steep decrease that leveled off at CFs > 0.6. When removing the RL component by simulating 3T3_*HR*_ cell activation, the moderate decay of θ at CFs < 0.3 remained largely unchanged. On close inspection, conduction velocities during simulated illumination were in fact slightly lower than those obtained in dark conditions, which can be explained by the loss of the support by supernormal conduction that is afforded, in the dark, by the depolarizing influence of 3T3_*HR*_ cells on coupled CMCs. The steep decline of conduction velocities at 0.3 < CF < 0.6 was explained by the equally steep decline of sodium current availability due to resistive loading of the CMCs by coupled 3T3_*HR*_ cells. Upon illumination, sodium current availability was fully restored, as were the simulated electrogram amplitudes. By contrast and in agreement with experiments, θ was only partially restored with the deficit being likely due to the continued presence of capacitive loading. That this was indeed the correct explanation was demonstrated by the finding that coupling of cells acting as pure capacitances slowed conduction in a manner indistinguishable from light-activated 3T3_*HR*_ cells. The isolation of the effect of resistive loading on propagation by subtracting the results obtained under light-on conditions (CL) from those obtained during light-off conditions (RL + CL) showed a slight acceleration of θ in the range 0 < CF < 0.3 reminiscent of NEC-induced supernormal conduction (Miragoli et al., 2006).

At maximal CFs tested, RL contributed ∼50% and CL ∼30% to overall conduction slowing (−80%). Differences in the CF dependence of the two components (monophasic for CL, multiphasic for RL) caused the two components to crossover at a CF of ∼0.5. Below this value, conduction slowing was dominated by CL, whereas above, RL became increasingly important.

The comparison of experimental and simulation results showed convergence for all parameters investigated including changes in electrogram amplitudes and the dependence of conduction velocities on CFs in the presence and absence of resistive loading. Of particular interest for this study, fitting the CL-dependent conduction slowing to a power function unveiled θ to be inversely proportional to *C*_*m*_^–0.99^ (experimental data; 95% confidence interval = *C*_*m*_^–1.42^ to *C*_*m*_^–0.57^) and *C*_*m*_^–0.71^ (simulation: 95% confidence interval = *C*_*m*_^–0.73^ to *C*_*m*_^–0.68^). These experimental results, even though displaying a broad confidence interval because of data scattering, provide for the first time a “wet” verification of the longstanding theoretical prediction that membrane capacitance determines cardiac θ according to a power law with an exponent between −1/2 and −1.

To test whether findings obtained with 3T3_*HR*_ cells are relevant for other types of NECs as well, results were compared to HR-transduced cardiac myofibroblasts (MFB_*HR*_). The comparison showed that, at equal levels of depression of conduction in the dark, light activation of HR caused similar increases of θ with the resistive and capacitive loading components of 3T3_*HR*_ cells matching those of MFB_*HR*_ cells (Supplementary Figure S7). This suggests that the conclusions drawn from 3T3_*HR*_ cell experiments in the present study are likely valid for other NECs that exhibit a depolarized phenotype and establish heterocellular electrotonic coupling with CMCs.

## Limitations of the Study

The main limitation of this study in respect to describing the dependence of θ on membrane capacitance refers to the problem of obtaining exact values of the cell membrane areas of CMCs and 3T3_*HR*_ cells. Whereas the CMC strands were formed by continuous cell monolayer, taking the area of these strands as a measure of CMC capacitance likely represented an underestimate because CMCs forming these monolayers are known to overlap to a certain extent. Similarly, the determination of the cell area covered by 3T3_*HR*_ cell based on their eYFP fluorescence was likely underestimating the true area because eYFP fluorescence tends to escape detection in fine and flat extensions of the fibroblastic cells. While obtaining the ratio of the two parameters as used in the study likely alleviated this problem to some extent, the power law coefficient describing the dependence of conduction on membrane capacitance is subject to some uncertainty as illustrated also by the wide confidence interval.

Linked to the problem of an exact determination of the membrane area of 3T3_*HR*_ cells is the presence of endogenous myofibroblasts that, similar to 3T3_*HR*_ cells, induce slow conduction in a cell density–dependent manner (Miragoli et al., 2006). Underlying mechanisms are identical; that is, myofibroblasts display, compared to CMCs, a depolarized phenotype and, once electrotonically coupled to the latter, represent a capacitive and resistive load. In the context of our study, myofibroblasts will add membrane capacitance to the system, and their RL will persist during illumination as they are devoid of the optogenetic actuator. In previous works, the baseline myofibroblast content of our cell cultures was estimated to be less than 10% (Rohr et al., 1991; Miragoli et al., 2006). To investigate the contribution of these non-actuated cells to conduction slowing in the presence of 3T3_*HR*_ cells, we performed additional simulations (Supplementary Figure S8) in which we added 10% of non-excitable and non-actuated cells to the 3T3_*HR*_-CMC strands. The simulations showed that the presence of these myofibroblast surrogates essentially shifts the relationship between θ and the coverage by 3T3_*HR*_ cells by 0.1 CF unit to the left. Important to note in the context of this study is that the shift did not substantially modify the effect of capacitive loading and its power-law relationship to θ.

## Conclusion

This study experimentally untangles the relative contributions of resistive loading (aka depolarization) and capacitive loading to conduction slowing induced by NECs that are electrotonically coupled to CMCs. At low numbers of NECs, conduction slowing is exclusively related to capacitive loading. At moderate numbers of NECs, the rate of conduction slowing increases based on the combined effects of resistive loading causing sodium current inactivation and the further increase of capacitive loading. Both effects are balanced when the total membrane area of coupled NECs reaches ∼50% of the membrane area of CMCs. Beyond this value, resistive loading becomes the prominent factor in conduction slowing. Unveiling this cell density–dependent change of mechanisms dominating NEC-induced conduction slowing contributes to the general understanding of the biophysical mechanism underlying NEC-dependent modulation of conduction. Appreciating the importance of both mechanisms is likely relevant, for example, for cardiac stem cell therapies with communication-competent cells that may act proarrhythmic (Almeida et al., 2015) not only because of constituting an RL (Smit and Coronel, 2014) but also, as demonstrated in this study, by inducing slow conduction based on capacitive loading. Finally, the study provides for the first time experimental data describing the dependence of cardiac impulse conduction on membrane capacitance. Experimental data are in line with the theoretical prediction that cell membrane capacitance affects θ in cardiac tissue according to a power law with an exponent ranging from −1/2 to −1.

## Data Availability Statement

The datasets generated for this study are available by request to the corresponding author.

## Ethics Statement

The animal study was reviewed and approved by the State Veterinary Department of the Canton Bern, licence BE27/17.

## Author Contributions

SR conceived the study. SD designed and performed the 3T3 cell experiments, analyzed and interpreted the data, and wrote part of the methods section. AB performed the simulations and wrote part of the methods section. SM developed the optogenetically modified cardiac myofibroblasts and conducted the respective experiments. CD developed the MEA system. Computational and experimental work was supervised by JK and SR who also wrote the manuscript. All authors read and approved the submitted version of the manuscript.

## Conflict of Interest

The authors declare that the research was conducted in the absence of any commercial or financial relationships that could be construed as a potential conflict of interest.
