# Supplementary material for: The Role of Membrane Capacitance in Cardiac Impulse Conduction: An Optogenetic Study With Non-excitable Cells Coupled to Cardiomyocytes
Source: Front Physiol. 2020 Mar 26;11:194. doi: 10.3389/fphys.2020.00194 (PMC7113375; doi:10.3389/fphys.2020.00194)
Supplement: Supplementary file 1 [file Data_Sheet_1.pdf]

Supplementary Material

1. Layout of MEA substrates

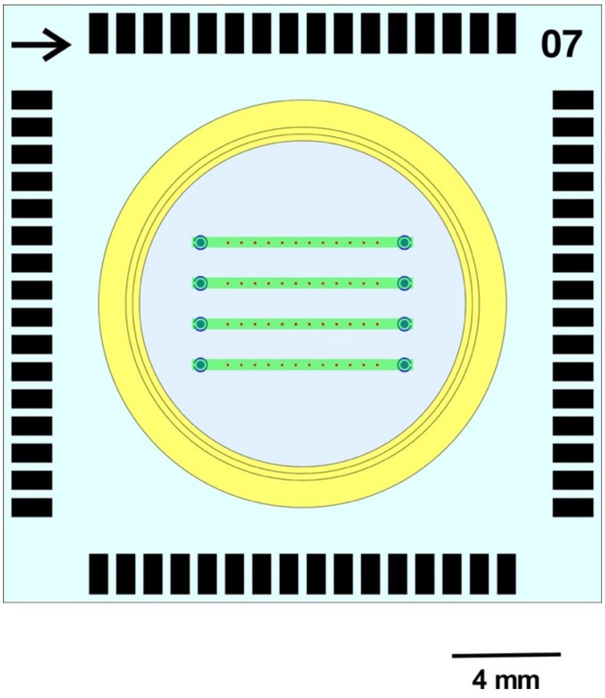

Supplementary Figure S1: Layout of MEA substrates. Yellow ring: culture cylinder. Red dots: recording electrodes. Blue structures: stimulation dipoles. Green bands: position of strand preparations.

2. Determination of the $3T3_{HR}$ cell coverage factor

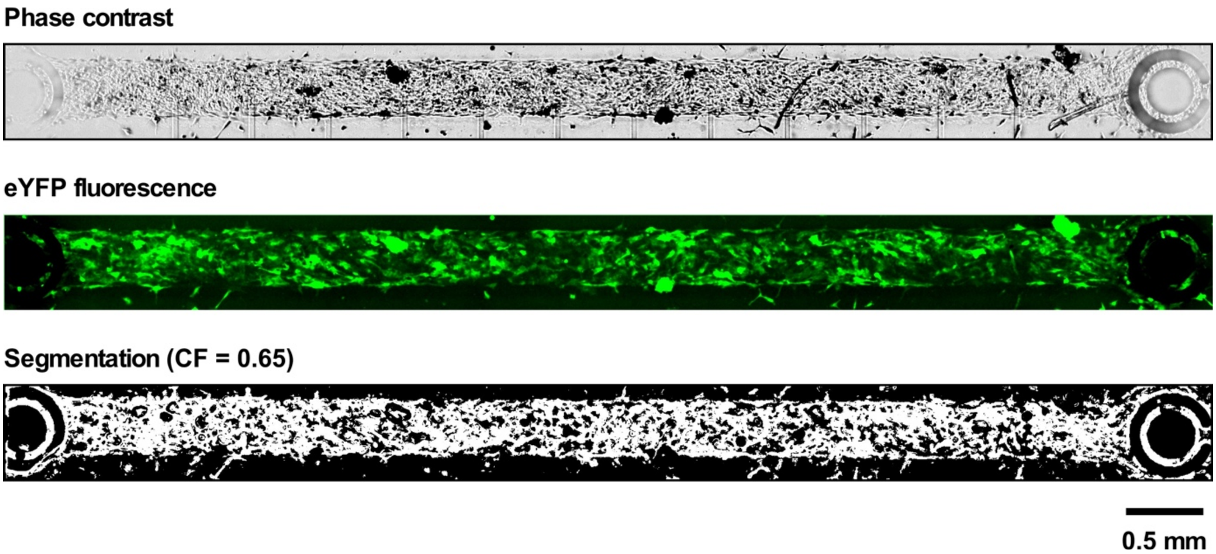

Supplementary Figure S2: Morphology of a strand preparation as used in the MEA experiments. Upper panel: bright field image of the preparation. Middle panel: fluorescence image of $3T3_{HR}$ cells growing on top of the CMCs and expressing eYFP. Bottom panel: segmentation of eYFP fluorescence yielded a coverage factor (CF) of 0.65 for this particular preparation.

3. Halorhodopsin expression vs. I_{HR} current density

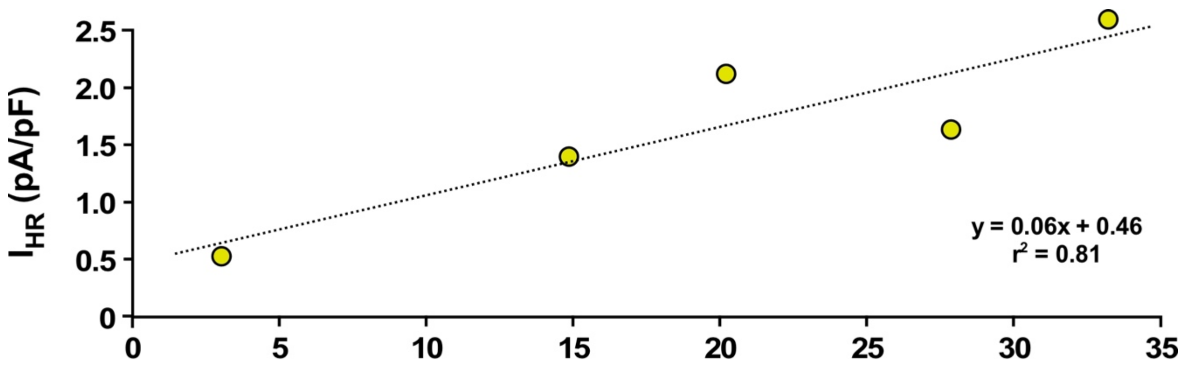

Supplementary Figure S3: Dependence of light activated I_{HR} density on the expression of eNpHR3.0-eYFP. Reporter (eYFP) related fluorescence intensity is given in arbitrary units.

4. Kinetics of the light-induced hyperpolarizing response of $3T3_{HR}$ cells

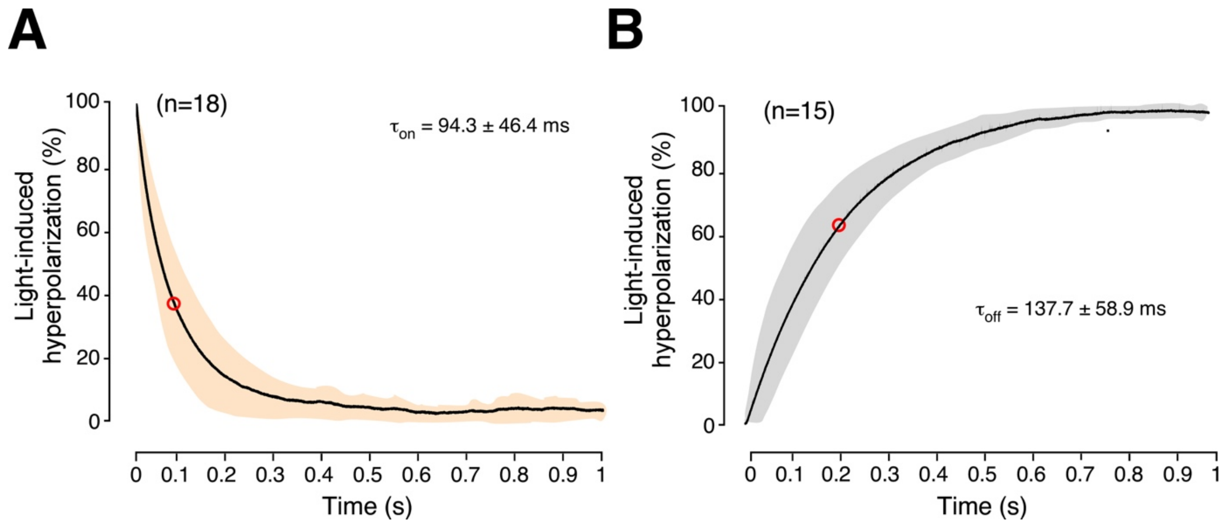

Supplementary Figure S4: Kinetics of HR activation and deactivation. **(A)** Time course of hyperpolarization upon light-activation of $3T3_{HR}$ cells (τ_{on} : activation time constant). **(B)** Time course of return to depolarized values after stopping illumination (τ_{off} : deactivation time constant). Red circles indicate τ. Values refer to the mean +/- SD of the normalized data.

5. CMC-$3T3_{HR}$ gap junctional coupling: connexin43 (Cx43)

Immunocytochemical evidence for gap junctional coupling between CMCs and $3T3_{HR}$ cells was obtained from low density co-cultures of the respective cell types. Culturing procedures followed those described in the main article. CMCs were seeded onto collagen type IV (Sigma) coated glass coverslips at 160 cells/mm^2 with 3T3HR cells being added at 30 cells/mm^2 24 h later. After a further 48 h of incubation, preparations were washed three times with Hanks' balanced salt solution (Lonza, Switzerland) followed by fixation with 2% paraformaldehyde for 5 min. After another washing step with PBS (AppliChem, Germany), preparations were incubated for 20 min with blocking buffer (PBS containing 20% goat serum) before being exposed, after a further washing step (PBS), for 2 h to the Cx43 antibody (mouse monoclonal, Millipore) dissolved in PBS containing 1% goat serum and 0.15% triton X-100 (BioRad, Switzerland). After washing, preparations were incubated for 20 min with the secondary antibody (AlexaFluor 488, goat anti-mouse, Life Technologies, Europe) and DAPI (Life Technologies, Europe). This step was followed by washing and mounting (Fluoroshield, Sigma). All steps were performed at room temperature. The preparations were imaged at 64x on an inverted microscope equipped for epifluorescence (Zeiss, Axiovert 200) using a high sensitivity camera (Spot RT, Diagnostic Instruments). Image analysis was performed with ImagePro (Media Cybernetics).

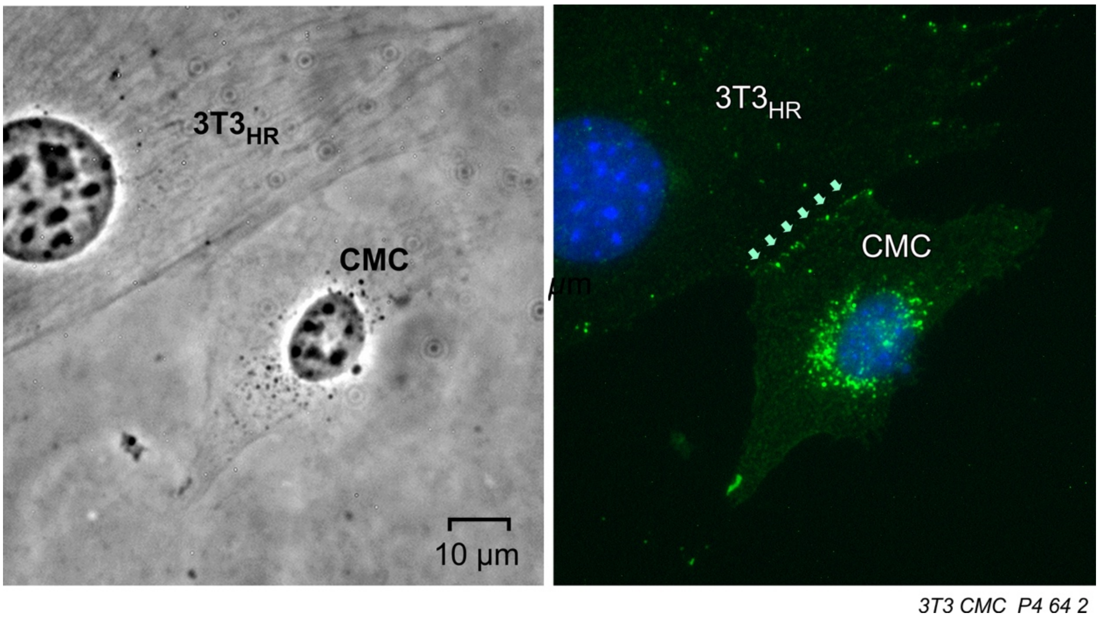

Supplementary Figure S5: Phase contrast (left panel) and immunofluorescence (Cx43; right panel) image of the contact site between a $3T3_{HR}$ cell and a cardiomyocyte (CMC). The lateral site of contact is decorated with fine-punctate Cx43 fluorescence (green arrows) that suggests presence of heterocellular gap junctional coupling. Blue: DAPI staining of the nuclei.

6. Simulated impulse conduction in presence of weak heterocellular coupling

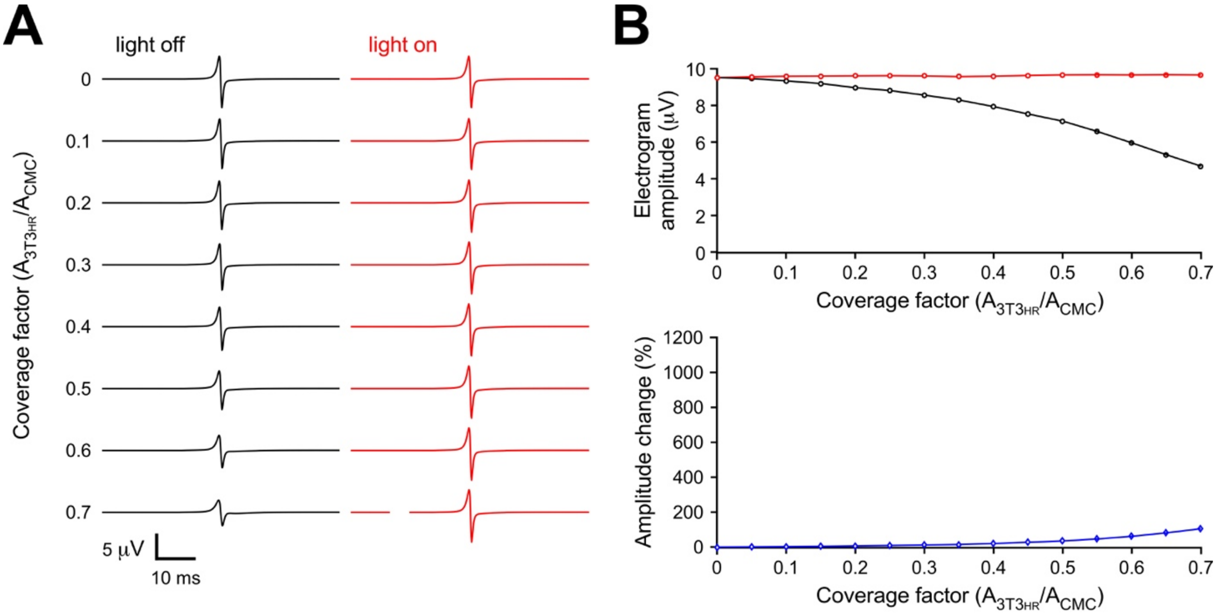

Supplementary Figure S6. Simulated extracellular electrograms for increasing coverage factors and a reduced CMC-$3T3_{HR}$ cell coupling conductance of 1.2 nS. **(A)** Simulated electrograms under dark conditions (light off, black) and during light stimulation (light on, red). **(B)** Corresponding electrogram amplitudes (top) and relative amplitude changes (bottom) upon illumination vs. coverage factor.

7. Partial rescue of slow conduction by optogenetic actuation: Comparison of $3T3_{HR}$ cells and HR-transduced cardiac myofibroblasts (MFB_{HR})

Based on the finding that cardiac myofibroblasts (MFBs) decrease conduction velocity (θ) in strands of CMCs in a cell-density dependent manner upon establishing heterocellular gap junctional coupling (Miragoli et al. Circ Res. 2006;98:801–810), we used HR-transduced cardiac myofibroblasts in pilot experiments to investigate the relative contributions of resistive vs. capacitive loading on conduction slowing before switching to the $3T3_{HR}$ model. The availability of data from 9 independent experiments with CMC-MFB_{HR} preparations permits conducting a post hoc comparison of the two cell types in regard to their resistive and capacitive loading properties. The experimental approach used in the context of MFB_{HR} cells was identical to the methods described for the $3T3_{HR}$ cell experiments. Differences concerned the type of preparation (monolayer CMC cultures vs. CMC strands) and the circumstance that coverage factors (CFs) were not determined in the CMC-MFB_{HR} preparations. The latter difference prohibits a direct comparison of the two cell types on the basis of the CF. However, results can be compared in regard to the HR-activation induced increase of θ to 'θ_{light}' at any given level of conduction slowing measured in the dark (θ_{dark}). The results of a single experiment with a CMC-MFB_{HR} preparation is shown in Fig. S7A. Similar to the $3T3_{HR}$ preparations (Fig. 2B of the manuscript), θ increases during HR activation (from 231 mm/s to 285 mm/s) in the MFB_{HR} coated CMC preparation while illumination has no significant effect on θ in CMC control preparations. As shown in Fig. S7B, the light-induced increase of θ is inversely related to θ_{dark} for both MFB_{HR} (left panel) and $3T3_{HR}$ coated CMC preparations (right panel). When correlating the deficit of θ of $3T3_{HR}$-CMC preparations in respect to $\theta_{control}$ of CMC preparations (358 ± 35 mm/s, n=8) for θ_{dark} and θ_{light}, values obtained with $3T3_{HR}$ and MFB_{HR} cells largely

overlap (Fig. S7C). This suggests that the contribution of resistive loading to conduction slowing is similar for $3T3_{HR}$ and MFB_{HR} cells. Moreover, because the θ_{light} deficit is a measure of capacitive loading, the extent thereof is similar for both cell types as well. Taken together, the data overlap suggests that the conclusions drawn on the basis of the $3T3_{HR}$ cell experiments are likely valid for cardiac myofibroblasts as well.

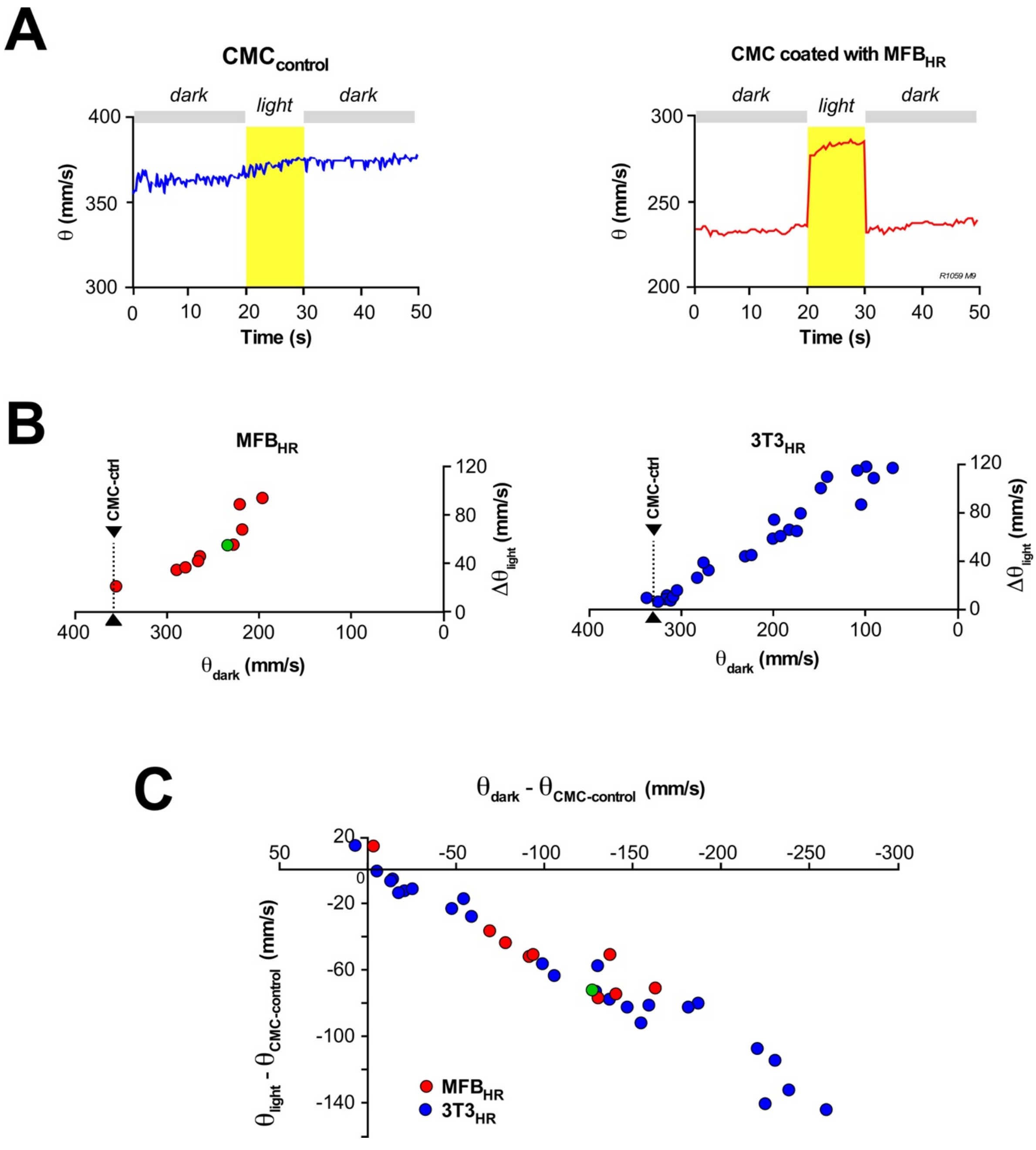

Supplementary Figure S7. Comparison of modulation of θ by MFB_{HR}- and $3T3_{HR}$ cells. **(A)** Conduction velocities (θ) in a control CMC monolayer (left panel) and a CMC monolayer coated with MFB_{HR} cells during application of HR-activating light (right panel). **(B)** HR-activation induced increase of θ as a function of θ measured in the dark for MFB_{HR}- (left panel) and $3T3_{HR}$ preparations (right panel). **(C)** Conduction slowing induced by MFB_{HR}- and $3T3_{HR}$ cells (abscissa) is similarly restored by light-activation of HR (ordinate) in both types of preparations. Green data points shown in panels B and C correspond to the experiment shown in panel A.

8. Interpretation of $3T3_{HR}$ cell data in presence of endogenous, non-actuated myofibroblasts in the cell strands

Despite undertaking efforts to reduce the content of endogenous myofibroblasts (MFBs) by differential pre-plating, our CMC cell cultures contain, on average, 9% MFBs (Miragoli et al. Circ Res. 2006;98:801–810). Because MFBs, like $3T3_{HR}$ cells, contribute to conduction slowing but are not actuated by light, they affect baseline conduction by inducing a slight depolarization of coupled CMCs (resistive load) and by contributing to the capacitive load. At first approximation and under the assumption that the electrophysiological characteristics of MFBs are similar to those of $3T3_{HR}$ cells, increasing the coverage factor by 10% with non-actuated MFBs will shift the results shown in Figure 4 of the main article by an equal amount to the left.

To ascertain this assumption, we performed a simulation in which we assumed a 10% baseline level ($p_{baseline}$) of non-excitable, non-actuated cells, representing the endogenous myofibroblasts content. We assumed that these additional cells have an ion current repertoire comparable to $3T3_{HR}$ cells except for I_{HR}, which was set to 0 to take into account that these cells were not actuated by light. We also assumed that these additional cells are situated on top of the cardiomyocytes and that the coupling between these cells and the myocytes is similar to that between $3T3_{HR}$ cells and CMCs. In the simulations, the coverage factor was set to CF_{set} (= experimentally determined CF) + $p_{baseline}$ (=0.1) and the ion currents of the additional non-excitable cells and the $3T3_{HR}$ cells were scaled accordingly.

The results of these simulations for a coupling conductance between cardiomyocytes and non-excitable cells of 72 nS are shown in Fig. S8. Compared, to simulations without endogenous non-excitable, non-actuated cells (Fig. 4B of the main article and Fig. S8A), the relationship between θ and the coverage by $3T3_{HR}$ cells (CF_{set}) appears similar in the additional presence of 10% non-actuated cells, and the curves largely overlap upon a shift by 0.1 along the abscissa (Figure S8B). This result suggests that the small baseline myofibroblasts content of cardiomyocyte cultures can be taken into account by shifting the experimental data along the x-axis. Of note, the effect of capacitive loading remains almost identical.

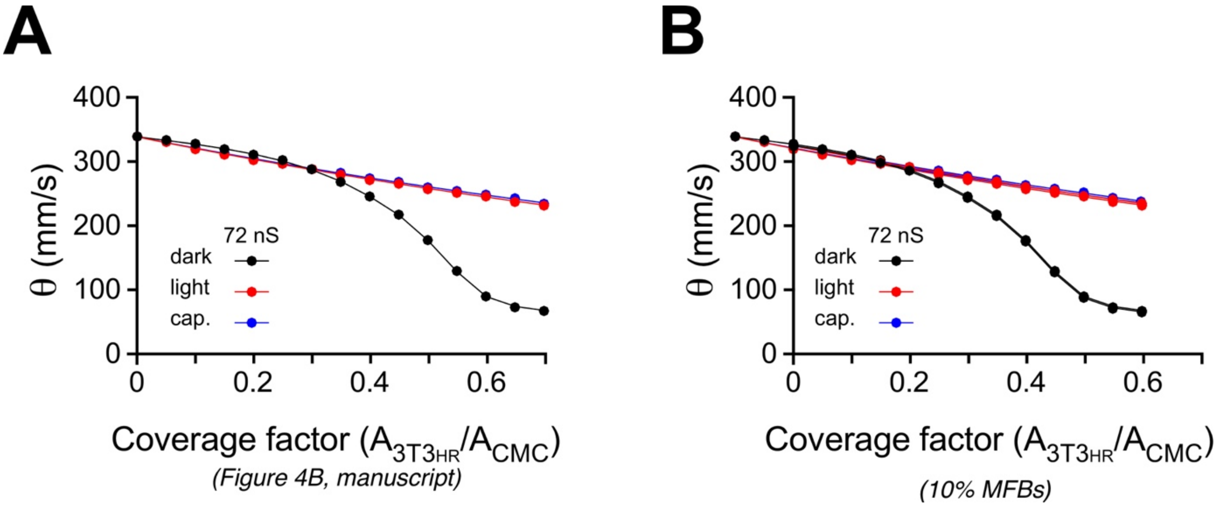

Supplementary Figure S8. Conduction in the added presence of non-actuated cells (MFBs). (A) Dependence of θ on the density of $3T3_{HR}$ cells (Figure 4B of the main article). **(B)** Dependence of θ on the density of $3T3_{HR}$ cells in presence of 10% non-actuated cells (MFBs). Values largely overlap with the data shown in (A) that are shifted by 0.1 to the left (lighter colors).
